# Aspiration-based coevolution of link weight promotes cooperation in the spatial prisoner's dilemma game

**DOI:** 10.1098/rsos.180199

**Published:** 2018-05-02

**Authors:** Chen Shen, Chen Chu, Lei Shi, Matjaž Perc, Zhen Wang

**Affiliations:** 1 School of Statistics and Mathematics, Yunnan University of Finance and Economics, Kunming, Yunnan 650221, People's Republic of China; 2 Faculty of Natural Sciences and Mathematics, University of Maribor, Koroska cesta 160, 2000 Maribor, Slovenia; 3 CAMTP—Center for Applied Mathematics and Theoretical Physics, University of Maribor, Mladinska 3, 2000 Maribor, Slovenia; 4 Complexity Science Hub, Josefstädterstraße 39, 1080 Vienna, Austria; 5 School of Mechanical Engineering and Center for OPTical IMagery Analysis and Learning (OPTIMAL), Northwestern Polytechnical University, Xi'an 710072, People's Republic of China

**Keywords:** coevolution, cooperation, aspiration

## Abstract

In this article, we propose an aspiration-based coevolution of link weight, and explore how this set-up affects the evolution of cooperation in the spatial prisoner's dilemma game. In particular, an individual will increase the weight of its link to its neighbours only if the payoff received via this interaction exceeds a pre-defined aspiration. Conversely, if the received payoff is below this aspiration, the link weight with the corresponding neighbour will decrease. Our results show that an appropriate aspiration level leads to a high-cooperation plateau, whereas too high or too low aspiration will impede the evolution of cooperation. We explain these findings with a comprehensive analysis of transition points and with a systematic analysis of typical configuration patterns. The presented results provide further theoretical insights with regards to the impact of different aspiration levels on cooperation in human societies.

## 1. Introduction

The emergence and maintenance of cooperation have long puzzled scientists of different disciplines due to the fact that cooperator often benefits defector at the cost of her/his own losses, which leads to the extinction of altruistic behaviour in the competition with self-interested players and thus gives rise to various social dilemmas. Evolutionary game theory has provided a powerful mathematical framework to solve this problem [1–3]. In particular, the prisoner's dilemma game (PDG) is a famous paradigm for cooperation. The original PDG depicts the pairwise interactions of players with two independent options: the two players must simultaneously decide whether to cooperate or to defect. Mutual cooperation (defection) leads to reward *R* (punishment *P*). If one player defects and the other cooperates, the traitor receives the temptation to defect *T*, while the latter is left with the sucker's payoff *S*. These payoff parameters satisfy *T* > *R* > *P* > *S* and 2*R* > *T* + *S*. According to such a ranking, one can see that defection is the best response for an individual. However, the best solution for the group is mutual cooperation. It creates an irreconcilable conflict between what is best for individual and what is best for group. In order to offset the above unfavourable outcome and enhance cooperation, many frameworks have been proposed, such as age [4–6], reputation [7–11], social diversity [12–18], asymmetric interaction [19–22], mobility [23–28], different update rules [29–33], as well as various network topologies [34–37], to name but a few [38–44].

The vast majority of the spatial models in previous literature have used static and unweighted networks. However, realistic social networks are not static; they can adaptively change in time. For example, an under-performance player will break a link and look for a more beneficial interaction with another one. Based on this fact, linking dynamics has attracted considerable attention, and the research about the effect of dynamic network on the evolution of cooperation has confirmed that cooperation can be boosted under certain circumstances (for a comprehensive discussion, see [45]). In spite of their great achievements, breaking a link with unsatisfied neighbour, however, seems a little rigorous. Breaking down the link with unsatisfied neighbour implies that except for looking for new neighbour, it is impossible to restore the connection with the former neighbour if the focal player regrets his decision. To overcome this shortcoming, people will reduce the probability to interact with undesired individuals and in turn increase the opportunity to interact with well-performed individuals. Here we consider game models on a more generous dynamically directed weighted network. In detail, individuals will reinforce their link weight only if the gains from the neighbours exceed their aspiration. Yet, as soon as the payoffs are lower than their aspiration, the link weight with the corresponding player will be weakened, in other words, both cooperator and defector have the same opportunity to get higher weight as reward when they meet certain conditions. Here the aspiration level can be interpreted as the degree of satisfaction of individuals with their neighbours.

In view of the above situation, we first present our aspiration-based coevolution model of the PDG where link weight will be strengthened as a reward once the focal player is satisfied with the gains from his neighbours, otherwise link weight will be weakened as the punishment. It is found that by such a simple coevolution rule, a plateau of high cooperation can be achieved with an appropriate aspiration level. The remainder of this paper is organized as follows. First, we describe our coevolution model. Next, we present the results, whereas lastly we summarize and discuss the main conclusions.

## 2. Model

Here we consider the PDG, which is *R* *=* 1*, P* = *S* = 0, and *T* *=* *b*, where *b* (1 < *b* ≤ 2) represents the temptation to defect. As for interaction network, we choose the regular square lattice with von Neumann neighbourhood and periodic boundary conditions of size *L* × *L*. Initially each player *x* is designated either as a cooperator (*s_x_* = *C*) or defector (*s_x_* = *D*) with equal probability. Link weight is introduced into the model in the following way: each edge linking node *x* and *y*, at the beginning, is assigned the same weight value *w_xy_* = 1, which, however will adaptively change in accordance with the interaction (we assume the range of link weight falls into [0, 2] in this paper).

At each time step, each player *x* first acquires his payoff *p_xy_* by playing the game with his neighbour *y*. If the payoff *p_xy_* from neighbour *y* exceeds his aspiration, player *x* will reinforce the relationship with player *y* in order to gain more benefits for the next interaction*,* otherwise player *x* will weaken the link weight to reduce his losses. This process can be described as the following equation:

2.1 $$\left\{ \begin{array}{ll} w_{xy}=w_{xy}+\Delta , & \mathrm{if}\;P_{xy}\geq A\\ w_{xy}=w_{xy}-\Delta \; & \mathrm{if}\;P_{xy^{\prime }}<A. \end{array}\right.$$

Here we define *A* = *α* * *b* as individual's aspiration level. Obviously, if aspiration is too large, all players will be dissatisfied with their neighbours and thus all the link weight will decrease to zero. In this case, players update their strategy just like a coin toss. To avoid this extreme situation, the maximum of *α* is fixed at 0.99 in our simulation. Besides, we call Δ as players' tolerance degree. Δ = 0 will lead the system to the traditional case. The larger the value of Δ, the lower the degree of individuals' tolerance. Then, combing link weight and the aforementioned payoff *P_xy_*, player *x* will get his accumulated utility as follows:

2.2 $$F_{x}=\underset{y\in \Omega }{\sum }w_{xy}*P_{xy},$$

where *Ω* is the set of neighbours of player *x*. When the focal player *x* updates his strategy, it will pick up randomly one neighbour *y*, who also gets his fitness *F_y_* in the same way, and decides whether to adopt the strategy of player *y* with a probability given by Fermi function as follows:

2.3 $$w=\frac{1}{1+\exp((F_{x}-F_{y})/K)},$$

where *K* denotes the amplitude of noise or its inverse the so-called intensity of selection. In this paper, we fix the value of *K* to be *K* = 0.1 [46–48].

Simulation results presented below were obtained for populations comprising 100 × 100 individuals. Besides, the key quantities of the fraction of three strategies are determined within the last 10^3^ full MCS over the total 10^4^ steps. Moreover, to avoid additional disturbances, the final results are averaged over up to 10 independent realizations for each set of parameter values in order to ensure suitable accuracy. Besides, we have also conducted the asynchronous updating method, which produces qualitatively similar results. To be simple, we do not show them here.

## 3. Results

**Figure 1.**
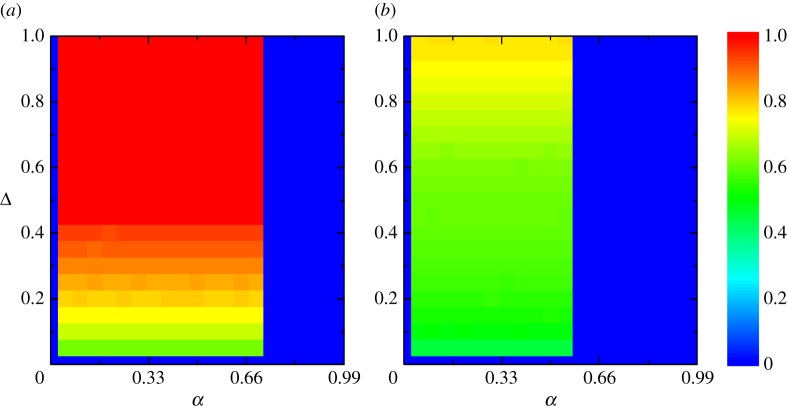
Colour-code (see bar on the right) fraction of cooperation on the *α* – Δ parameter plane for *b* = 1.4 (*a*) and *b* = 1.8 (*b*).

Figure 1 presents the colour map encoding the fraction of cooperation *ρ_c_* on the *α*– Δ parameter plane for different values of *b*. Evidently, there exists an intermediate aspiration level, at which the cooperation can be optimally promoted. While too small or too large aspiration will impede the evolution of cooperation and even lead to the complete pure D phase. Additionally, the value of phase transition point and the maximum cooperation level will decrease with increasing *b*. While for the impact of the degree of tolerance Δ, it is obvious that the faster the response to the opponent (the larger the value of Δ), the higher the level of cooperation. For large *b* (figure 1*b*), the promotion effect of Δ becomes less striking. In fact, increasing Δ induces the increasing of the average link weight, which plays a crucial role in promoting cooperation as shown in [49,50]. In a word, our aspiration-based coevolution set-up can truly promote cooperation. An intermediate aspiration level can optimally boost cooperation; however, just slightly exceeding the transition point, cooperation disappears, and in what follows we will give a further understanding about the above results.

**Figure 2.**
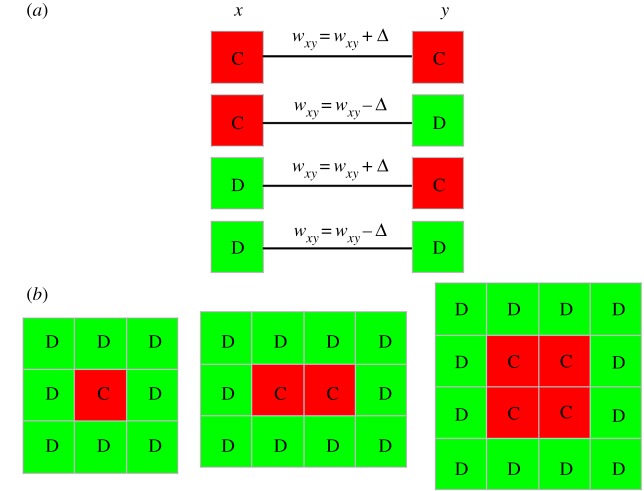
(*a*) The edge weight evolution in the PDG. The edge types between any two players can be divided into four relations: C-C, C-D, D-C, D-D, respectively. (*b*) Special configuration patterns reveal their potential to expand into the territory of defectors.

Rough interfaces, which separate domains of cooperators and defctors, as the evolution along them has been identified crucial for deciding who is the winner on the evolutionary dynamics. It is next of significant interest to further examine the spatial configuration patterns. Since our coevolution set-up evokes a dynamic change of link weight, we first analyse how link weight fares for different types of strategy pairs under different aspiration levels in figure 2*a*. According to the definition of parameters in PDG, we can see that players can obtain *b*, 1, 0, 0 units from DC, CC, CD or DD links, respectively. When aspiration is moderate, the weights of CD and DD links always decrease, and thus the evolution trend mainly depends on CC and DC links. When *A* < 1, namely, *α* < 1/*b*, both CC and DC links are satisfied with their performance and hence the link weight *w*_DC_ and *w*_CC_ will get an increase. While *A* > 1 (*α* > 1/*b*) only renders DC links satisfied with their performance and thus *w*_DC_ will get an increase, the other three types are not. In what follows, we will take a closer look at how aspiration level can affect the evolution of cooperation.

Figure 2*b* presents some special configuration patterns. When 0 < *α* < 1/*b*, A cooperator is satisfied with his cooperative neighbours and will reinforce the weight with C neighbours. At the same time, he is dissatisfied with his defective neighbours and will weaken the weight with D neighbours. In this way, we can calculate his utility as *F*_C_ = 0, $F_{C}=1+\Delta$, and $F_{C}=2+2*\Delta$, from left to right, respectively. As for the utility of defective player, we can also get his utility in the same way as $F_{D}=b+\Delta$ for these configuration patterns. By comparing the utility of cooperation and defection of these configuration patterns, we can easily conclude that cooperators will be wiped out by defectors for the left and middle panel, while cooperators can effectively spread their strategies to the whole network for the right panel. That is to say, as soon as the configuration like right panel is formed during the evolutionary dynamics, cooperation can be extensively enhanced. When *α* > 1/*b*, DC links are satisfied and other three types are not. That is to say, defectors can gain more payoffs for the appropriate aspiration, and cooperators will obtain lower payoffs for the decreased link weight. In fact, the condition *α* < 1/*b* renders all cooperators worthy of the reward, which is caused by an increased link weight between two cooperators, and thus introduces an enhanced network reciprocity into the system. The evolution of cooperation then proceeds with the support of this enhanced network reciprocity. However, the condition *α* > 1/*b* makes defectors rewarded and cooperators punished, which weakens the network reciprocity and leads to the demise of cooperation. What is more, one can find that in order for the spread of cooperation, two conditions must promise. Firstly, forming clusters (at least two C neighbours surrounded him), which ensures basic advantages over defectors in terms of payoffs. Secondly, cooperators cannot set too high aspirations such that their satisfactions can be easily achieved. Only with the fulfilment of the above two conditions can cooperative strategy spread to the whole network.

**Figure 3.**
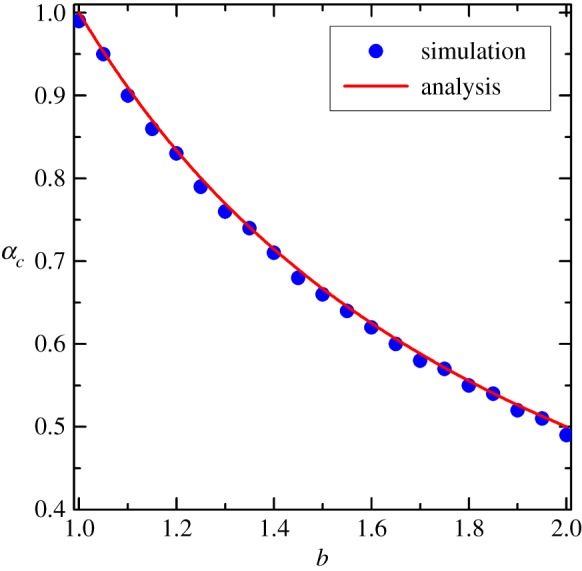
The relationship between the aspiration threshold *α_c_*, where cooperation dies out, and the temptation to defect *b*. We fixed Δ = 0.5.

From the above discussion, we can see the phase transition point, where cooperation dies out, seems equal to 1/*b*. To verify this guess, figure 3 depicts the relationship between the phase transition point *α_c_* and temptation to defect *b* for simulation results and theoretical analysis results. Evidently, theoretical analytical results are quantitatively consistent with simulations although tiny deviation exists, and the hasher the strength of social dilemma, the smaller the value of transition point.

**Figure 4.**
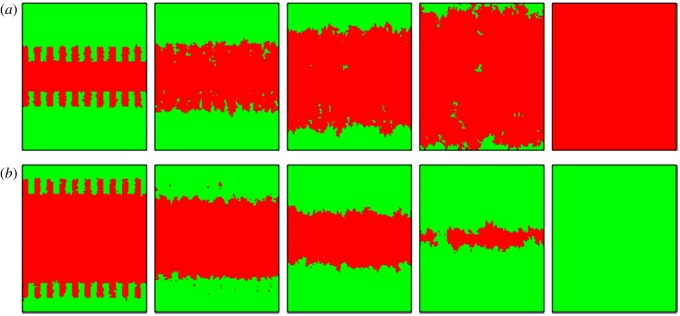
Evolution of a prepared initially rough interface, as obtained for *α* = 0.71 (*a*) and *α* = 0.72 (*b*). Different from the random distribution, there is a prepared initial state where interfaces separate domains of cooperators and defectors. Obviously, just a tiny increase in *α* exceeding *α_c_* (*α_c_* = 0.71 in this case) leads network reciprocity to be weakened and thus accelerate the demise of cooperation. The snapshots were taken at MCS = 0, 10, 40, 70, 9999 for (*a*), and at MCS = 0, 10, 30, 60, 9999 for (*b*). In both cases the temptation to defect *b* and the degree of tolerance were fixed to 1.4 and 0.5, respectively. What is more, cooperators and defectors are denoted by red and green.

Finally, in order to provide direct visual evidence that appropriate aspiration level will elevate cooperation while a tiny increase will decrease cooperation. Figure 4 depicts the characteristic spatial patterns. Different from random distribution, we start with rough interfaces, separate the domains of cooperators and defectors, to explore the evolution of cooperation. For *α* = 0.71 (figure 4*a*), where the aspiration level is moderate and both cooperation and defection are rewarded, cooperators can easily expand their territory, and their expansion makes the interface become more smooth (second column). This in turn further strengths the network reciprocity and finally results in complete C dominance. For *α* = 0.72 (figure 4*b*), when the aspiration level slightly exceeds the transition point, the evolution is significantly different. Here although the large and compact cooperative clusters are formed initially, the cooperators are inevitably exploited by defectors until pure D phase. The result of this process actually lies in enhanced network reciprocity and weakened network reciprocity as we mentioned above.

## 4. Conclusion

In [51–56], the rewiring of existing links was recognized as being very beneficial to the evolution of cooperation, and the growth of a network had a positive impact on the evolution of cooperation. Different from these dynamical networks, we adopt static weighted network in combination with the individual's aspiration, but we also find that intermediate aspiration level boosts cooperation. Moreover, there exists phase transition point in our results, which is also explicated in our work. In a sense, our work may enrich the content of evolutionary game dynamics especially aspiration-based evolution and weighted networks.

To conclude, we have introduced an aspiration-based coevolution set-up into the PDG, where a player reinforces his link weight only if the payoff from his neighbour exceeds his aspiration. Yet, as soon as the payoff is lower than his aspiration, the link weight with corresponding player will be weakened. Interestingly, appropriate aspiration level leads to a plateau of high cooperation; too large or too small aspiration, however, will impede the evolution of cooperation. For a comprehensive understanding, we have also given a further explanation of the above results and phase transition points existing in our simulation by means of configuration patterns. Finally we hope this work can inspire more studies for resolving the social dilemma, especially from the viewpoint of weight static networks and the individuals' aspiration level. Finally, we hope our work can be applied to other fields, especially in engineering [51–53].

## Data accessibility

The computer codes used to produce the data in the figures are available at the Dryad Digital Repository: https://doi.org/10.5061/dryad.sq8gc87 [57].
